# Determination of Specificity and Sensitivity of Anti-RA 33 in Diagnosis of Early Rheumatoid Arthritis

**DOI:** 10.5539/gjhs.v6n4p292

**Published:** 2014-05-28

**Authors:** Mahin Lashkari, Akram Noori, Fatemeh Hajiimanouchehri, Sonia Oveisi, Amir Mohammad Kazemifar

**Affiliations:** 1 Metabolic Diseases Research Center, Qazvin University of Medical Sciences, Qazvin, Iran; 2 Pathology Department, Qazvin University of Medical Sciences, Qazvin, Iran

**Keywords:** rheumatoid arthritis, anti-RA-33, rheumatoid factor, diagnosis

## Abstract

**Background:**

Rheumatoid arthritis is a chronic inflammatory disease with uncertain etiology. It is characterized by symmetric polyarthritis in peripheral joints. Its diagnosis is based on clinical findings and serologic tests. However, its diagnosis is rarely conclusive in early course of the disease. So, its early diagnosis could be difficult. The present study was designed to evaluate the role of anti -RA33; an auto-antibody against RA33 in early diagnosis of the disease.

**Materials and Methods:**

forty three patients with RA who had been visited in a rheumatology clinic were randomly selected. Their disease has been diagnosed by a rheumatologist. They served as the case group. 55 persons were also chosen from healthy individuals who had attended in other clinic. They served as control. Their age and sex were matched with the case group. Anti-RA33 and RF titers were measured in their blood sample using standard methods.

**Findings:**

RF and anti-RA33 titers had significant correlation in the case group (p=0.015). Anti -RA33 test had 98% sensitivity, 20% specificity, 50% positive predictive value, and 90% negative predictive value.

**Conclusion:**

Anti -RA33 could have diagnostic and prognostic importance in diagnosis and evaluation of patients with RA, and its differentiation from other small joint disorders, particularly when the other serologic tests are negative.

## 1. Introduction

Rheumatoid arthritis (RA) is a chronic inflammatory disease with unknown etiology characterized by symmetric peripheral polyarthritis. RA is the most common form of chronic inflammatory arthritis. It often results in joint damage and physical disability. It is a systemic disease with a variety of extra-articular manifestations including fatigue, subcutaneous nodules, lung involvement, pericarditis, peripheral neuropathy, vasculitis, and hematologic abnormalities (McInnes & Schett, 2011).

The incidence of RA rises in ages between 25 and 55 years, and then reaches to plateau until the age 75, and afterward decreases. The presenting symptoms of RA typically result from inflammation of the joints, tendons, and bursas. The patients often complain from early morning joint stiffness that lasts more than 1 hour and improves with physical activity. The small joints of the hands and feet are the earliest involved joints. The initial pattern of joint involvement may be mono-articular, oligo-articular (less than 4 joints), or poly-articular (more than 5 joints); usually with symmetric distribution (Firth, 2011; Nyhäll-Wåhlin et al., 2011).

Diagnosis of RA is based on its typical signs and symptoms, with laboratory and radiographic confirmation. In 2010, a collaborative effort between the American College of Rheumatology (ACR) and the European League against Rheumatism (EULAR) leaded to revision of the 1987 ACR classification criteria for diagnosis of RA to improve its early diagnosis with the goal of identifying patients who would benefit from early performance of disease-modifying therapies (Thabet et al., 2012). However, some patients may remain undiagnosed, do not treat at appropriate time to reach the disease remission or low disease activity, and could face with adverse effects of more potent therapeutic agents or complications of the disease. Consequently, determination of a more conclusive test for diagnosis of RA is superlative.

The autoantibody reactivity defined as anti-RA 33 is against a component of the splicosome which is the heterogeneous ribonucleoprotein complex 36-kDa A_2_ protein. The antigen that is associated with mRNA involve in regulation of pre- mRNA splicing, mRNA transport and translation. The anti-RA 33 antibodies can be found in the tumor necrosis factor-transgenic mice that develop spontaneous arthritis. However, they may contribute in pathogenesis of diseases in a nonspecific manner. An exception is auto antibodies against hnRNP-A_2_, which appears to have some relevance with pathogenesis and diagnosis of RA. Hn RNP-A_2_ is found in skin, lymphoid tissues, brain, and reproductive organs with highest expression levels (Conrad et al., 2010). Anti-RA 33 antibodies occur in approximately one third of patients with RA. Its level remains normally constant in the course of the disease (Steiner & Smolen, 2002; Duskin & Eisenberg, 2005).

Because anti-hnRNPA_2_ is rarely seen in osteoarthritis, reactive arthritis, ankylosing spondylitis or psoriatic arthritis, it can be helpful for differential diagnosis of these diseases with RA, particularly in patients who have negative RF and/or ACPA tests. Specificity of Anti-hn RNPA_2_ antibodies is approximately 90% for RA, which is somewhat lower than the specificity of ACPA or Ig M-RF. Similar to RF and ACPA, anti-hnRNP-A_2_ antibodies may appear in the earlier stages of the disease. They do not correlate with Ig M-RF or ACPA and are also not associated with radiographic progression of the disease, but rather seem to characterize patients with more favorable prognosis (Nell et al., 2005). In some studies, it is reported that anti-RA33 could be seen in 1% of normal population. It has also been suggested that it can be measured in early stages of RA (van Boekel et al., 2002; Fritsch et al., 2002; Mediwake et al., 2001).

There are not sufficient reports about the value of anti-RA33 in diagnosis of RA. The present study was designed to evaluate the role of anti -RA33 in early diagnosis of RA in comparison with RF.

## 2. Materials and Methods

Forty three patients with RA whose disease was diagnosed by a rheumatologist were enrolled in the present study. They were randomly selected from a rheumatology OPD clinic in Qazvin city, Iran. They were assigned as case group. 55 apparently healthy individuals were also selected from attendants of an OPD clinic. They were assigned as control group. Inclusion criterion was RA which had been diagnosed by a rheumatologist. An exclusion criterion was suffering from rheumatologic disorders other than RA.

Details of clinical symptoms, the disease duration, morning stiffness, extra-articular findings, number of the affected joints and lab test results have been collected from the patients’ medical records. Furthermore, their age, gender and the level of education were informed.

The level of anti -RA33 was compared between the groups. It was measured by ELISA. The cut-off point curve of its level was drawn, in which the sensitivity, specificity, and negative and positive predictive values of the test have been calculated, as well as its age correlation, and its association with RF.

The study has been approved by local ethical committee of Qazvin University of Medical Sciences, Iran. All of the studied persons provided informed consent for participation in the study.

## 3. Results

The current study was performed on 98 persons included 43 patients and 55 normal subjects, among whom 21 were male. There was no significant difference between the groups in gender distribution, but in their age, most of the subjects in case group were 30–49 years old, and in their level of education, most patients had high school education or less, whereas a significant number of subjects in control group had higher education (Table 1).

**Table 1:** General characteristics of the studied persons in case and control group

variable		Case group n=43	Control group n=56	P value
sex	*male*	10 (47.6%)	11 (52.4%)	0.805
	*female*	33 (42.9%)	44 (57.1%)	
Age (years)	*Less than 30*	7 (35%)	13 (65%)	0.014
	*30-39*	10 (37%)	17 (63%)	
	*40-49*	10 (43.5%)	13 (56.5%)	
	*50-59*	7 (46.7%)	8 (53.3%)	
	*More than 60*	9 (100%)	0 (0%)	
The level of education	*No education*	6 (85.7%)	1 (14.3%)	
	*Primary school*	20 (69%)	9 (31%)	Less than 0.001
	*High school*	10 (38.5%)	16 (61.5%)	
	*college*	4 (50%)	4 (50%)	
	*Master of science*	3 (16.7%)	15 (83.3%)
	*higher*	0 (0%)	6 (100%)	

In patients group, the mean number of the affected joints was 14.79± 6.33. The mean duration of the disease was 10.53±10.29 months. The other findings about the case group were shown in Table 2. The RF titer was 57.16±67.35 in the case group. The mean concentration of anti RA33 was 28.34±16.21 IU/ml. As it has been demonstrated in Table 3, there was significant difference between the groups in concentration of RF and anti-RA 33.

**Table 2:** the disease characteristics in the case group

		Number (percent)
Morning stiffness	yes	42 (97.7)
	no	1 (2.3)
Duration of morning stiffness (hour)	Less than 1	1 (3.3)
	1	9 (30)
	1-2	17 (56.7)
	2	1 (3.3)
	More than 2	2 (6.6)
Extra-articular manifestation	yes	2 (4.7)
	no	41 (95.3)

**Table 3:** Comparison of RF and anti-RA33 concentrations and age between the studied groups

	Case group	Control group	T	P value
Age (year)	47.0±15.60	38.45±9.92	3.10	0.003
RF	57.16±67.35	17.81±38.81	3.45	0.001
Anti-RA33	28.34±16.21	21.66±7.31	2.47	0.016

There was significant and positive relationship between age and RF titer (P=0.001), while there was no significant relationship between age and anti-RA33 titer (Table 4). There was also significant and positive relationship between RF and Anti RA33 titers (P=0.015). As it can be seen in chart 1, if the cutoff of Anti-RA33 is set at 11.9, it has 98% sensitivity for recognizing patients with RA. The positive and negative predictive values of the test were 50% and 90%, respectively.

**Table 4:** Correlation between RF, anti-RA33 concentrations, and age between the studied groups

	age	RF	Anti-RA33
age	1	0.329 (p value less than 0.001)	*0.152 (p value=0.15)*
RF	-	-	0.249 (p value=0.015)
Anti-RA33	-	-	-

**Table 5:** Sensitivity and specificity of anti RA33 in various cut-off points

cut-off points	Sensitivity	1- specificity	P value
13.8	0.906	0.762	0.088
15.8	0.868	0.738	0.122
14.8	0.887	0.0762	0.166
11.9	0.981	0.833	0.020

**Figure 1:**
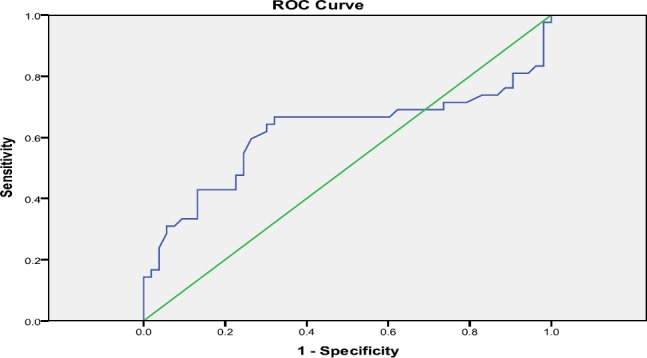
Sensitivity and specificity of anti-RA33

## 4. Discussion

In the present study, anti- RA33 concentration was 28.34±16.21 in patients with RA. The test had 98% sensitivity for recognizing patients with RA. Its positive and negative predictive values were 50% and 90%, respectively. There was significant difference in RF and anti-RA 33 titers between the studied groups.

Various auto antibodies may appear in the serum of the patients with RA. Some of them are more consistent and can be used as conclusive test for diagnosis of the disease, particularly, if they can be measured in the early course of the disease (Cordonnier et al., 1996).

In the study of Fritsch and his coworkers conducted on RA patients, anti-RA33 was positive in 73% of them (Fritsch et al., 2002). Its sensitivity and specificity for the disease recognition were 98% and 20% respectively, while positive and negative predictive values of the test were 55% and 90%, respectively (Fritsch et al., 2002). In another study performed on 10 patients with severe RF, 60% had positive anti-RA33. Among other 40 studied patients with non-erosive RA, RF was positive in 18% and anti-RA33 was positive in 60% of them (Chao et al., 2013). In another study conducted on patients with early RA, 40.8% had positive RF and 28.6% had positive anti-RA33. But, anti-RA33 was positive only 20% in those who had arthritis with unknown cause (Cordonnier et al., 1996). In the same study, sensitivity and specificity of the test were 85% and 28.6%, respectively. Its positive and negative predictive values were 82.3% and 32.7%, respectively (Cordonnier et al., 1996).

In the study of Al-Ani in Iraq conducted on 50 patients with established RA, 29 (58%) patients were anti-RA33 positive and 21(42%) patients were anti-RA33 negative (p<0.05). Sensitivity and specificity of anti-RA33 were 58% and 92.5%, respectively (Al-Ani, 2013).

The current study also showed that anti-RA33 test can be used as diagnostic clue for recognition of RA. The negative predictive value that attained in the present study, confirms the efficacy of the test in differentiation of RA from other similar diseases with small joints involvement. Moreover, as it is stated by other authors, anti-Ra33 antibodies are helpful in the diagnosis of RA patients who are anti-ccp and RF negative (Al-Ani, 2013).

The present study was performed on patients who were known cases of RA. If we were able to perform it in patients with early synovitis, we would be able to compare efficacy of RF and anti-RA33 in early diagnosis of RA, because patients with early synovitis who do not have criteria for diseases including RA, infections, spondyloarthropathies, and diagnosis must be by exclusion. Some trials revealed that 35-54% of patients did not meet for other diseases and were considered as undifferentiated arthritis (van Aken et al., 2003; Hülsemann & Zeidler, 1995). At present no standardized definition for early synovitis is accepted. Initial data suggest that the percentage of RA diagnosis will increase when the 2010 criteria for RA are applied (van der Linden et al., 2011). Therefore, autoantibodies could be helpful to differentiate patients with early synovitis, because autoantibody response occurres early during disorders before criteria are completed (Willemze, Ioan-Facsinay, & El-Gabalawy, 2008). This may be subject of future studies to evaluate effectiveness of the markers in early diagnosis of the disease.

## References

[ref1] Al-Ani M. M (2013). Comparison between anti-filaggrin, anti- RA33 and anti-cyclic citrullinated peptide antibodies in the diagnosis of rheumatoid arthritis in iraqi patients. Iraqi J Comm Med.

[ref2] Chao C, Liwei H, Quan C, Lan Z (2013). Clinical significance on antiperinuclear factor, anti-keratin antibody, anti-RA33 and anti-cyclic citrullinated peptide antibody in the diagnosis of rheumatoid arthritis. International Journal of Laboratory Medicine.

[ref3] Conrad K, Roggenbuck D, Reinhold D, Dörner T (2010). Profiling of rheumatoid arthritis associated autoantibodies. Autoimmunity reviews.

[ref4] Cordonnier C, Meyer O, Palazzo E, De Bandt M, Elias A, Nicaise P, Chatellier G (1996). Diagnostic value of anti-RA33 antibody, antikeratin antibody, antiperinuclear factor and antinuclear antibody in early rheumatoid arthritis: comparison with rheumatoid factor. Rheumatology.

[ref5] Duskin A, Eisenberg R. A (2010). The role of antibodies in inflammatory arthritis. Immunological reviews.

[ref6] Firth J (2011). Rheumatoid arthritis: diagnosis and multidisciplinary management. Br J Nurs.

[ref7] Fritsch R, Eselböck D, Skriner K, Jahn-Schmid B, Scheinecker C, Bohle B, Steiner G (2002). Characterization of autoreactive T cells to the autoantigens heterogeneous nuclear ribonucleoprotein A2 (RA33) and filaggrin in patients with rheumatoid arthritis. The Journal of Immunology.

[ref8] Hülsemann J. L, Zeidler H (1994). Undifferentiated arthritis in an early synovitis out-patient clinic. Clinical and experimental rheumatology.

[ref9] McInnes I. B, Schett G (2011). The pathogenesis of rheumatoid arthritis. New England Journal of Medicine.

[ref10] Mediwake R, Isenberg D. A, Schellekens G. A, Van Venrooij W. J (2001). Use of anti-citrullinated peptide and anti-RA33 antibodies in distinguishing erosive arthritis in patients with systemic lupus erythematosus and rheumatoid arthritis. Annals of the rheumatic diseases.

[ref11] Nell V. P. K, Machold K. P, Stamm T. A, Eberl G, Heinzl H, Uffmann M, Steiner G (2005). Autoantibody profiling as early diagnostic and prognostic tool for rheumatoid arthritis. Annals of the rheumatic diseases.

[ref12] Nyhäll-Wåhlin B. M, Turesson C, Jacobsson L. T. H, Nilsson J. Å, Forslind K, Albertsson K, Petersson I. F (2011). The presence of rheumatoid nodules at early rheumatoid arthritis diagnosis is a sign of extra-articular disease and predicts radiographic progression of joint destruction over 5 years. Scandinavian journal of rheumatology.

[ref13] Steiner G, Smolen J (2002). Autoantibodies in rheumatoid arthritis and their clinical significance. Arthritis Res.

[ref14] Thabet Y, Cañas F, Ghedira I, Youinou P, Mageed R. A, Renaudineau Y (2012). Altered patterns of epigenetic changes in systemic lupus erythematosus and auto-antibody production: is there a link?. Journal of autoimmunity.

[ref15] Van Aken J, Van Bilsen J. H. M, Allaart C. F, Huizinga T. W. J, Breedveld F. C (2003). The Leiden early arthritis clinic. Clinical and experimental rheumatology.

[ref16] Van Boekel M. A, Vossenaar E. R, Van den Hoogen F. H, van Venrooij W. J (2002). Autoantibody systems in rheumatoid arthritis: specificity, sensitivity and diagnostic value. Arthritis research.

[ref17] Van der Linden M. P. M, Knevel R, Huizinga T. W. J, van der Helm - van Mil A. H (2011). Classification of rheumatoid arthritis: comparison of the 1987 American College of Rheumatology criteria and the 2010 American College of Rheumatology/European League Against Rheumatism criteria. Arthritis & Rheumatism.

[ref18] Willemze A, Ioan-Facsinay A, El-Gabalawy H (2008). Anti-citrullinated protein antibody response associated with synovial immune deposits in a patient with suspected early rheumatoid arthritis. The Journal of rheumatology.

